# Enzyme-Treated *Zizania latifolia* Ethanol Extract Improves Liver-Related Outcomes and Fatigability

**DOI:** 10.3390/foods13111725

**Published:** 2024-05-31

**Authors:** Yu-Jin Ahn, Boyun Kim, Yoon Hee Kim, Tae Young Kim, Hyeyeong Seo, Yooheon Park, Sung-Soo Park, Yejin Ahn

**Affiliations:** 1 Dental Research Institute, School of Dentistry, Seoul National University, Seoul 03080, Republic of Korea; yuahn@snu.ac.kr; 2 Department of Smart-Bio, Kyungsung University, Busan 48434, Republic of Korea; boyunism@gmail.com; 3 R&D Center, BTC Corporation, Ansan 15588, Republic of Korea; kyh@btcbio.com (Y.H.K.); tykim@btcbio.com (T.Y.K.); 4 Department of Food Science and Biotechnology, Dongguk University, Goyang 10326, Republic of Korea; hyeyeong09@dongguk.edu (H.S.); ypark@dongguk.edu (Y.P.); 5 Department of Food Science and Nutrition, Jeju National University, Jeju 63243, Republic of Korea; 6 Research Group of Functional Food Materials, Korea Food Research Institute, Wanju-gun 55365, Republic of Korea

**Keywords:** *Zizania latifolia*, tricin, liver function, fatigability, clinical trial

## Abstract

Long-term hepatic damage is associated with human morbidity and mortality owing to numerous pathogenic factors. A variety of studies have focused on improving liver health using natural products and herbal medicines. We aimed to investigate the effect of enzyme-treated *Zizania latifolia* ethanol extract (ETZL), which increases the content of tricin via enzymatic hydrolysis, for 8 weeks on liver-related outcomes, lipid metabolism, antioxidant activity, and fatigue compared to a placebo. Healthy Korean adult males aged 19–60 years were randomized into ETZL treatment and placebo groups, and alcohol consumption was 24.96 and 28.64 units/week, respectively. Alanine transaminase, a blood marker associated with liver cell injury, significantly decreased after 8 weeks compared to the baseline in the ETZL treatment group (*p* = 0.004). After 8 weeks, the treatment group showed significant changes in the levels of high-density lipoprotein and hepatic steatosis index compared to the baseline (*p* = 0.028 and *p* = 0.004, respectively). ETZL treatment tended to reduce antioxidant-activity-related factors, total antioxidant status, and malondialdehyde, but there was no significant difference. In the multidimensional fatigue scale, ETZL treatment showed a significant reduction in general fatigue and total-fatigue-related values after 8 weeks compared to the baseline (*p* = 0.012 and *p* = 0.032, respectively). Taken together, the 8-week treatment of enzyme-treated *Zizania latifolia* ethanol extract demonstrated positive effects on liver-related outcomes, lipid metabolism, and mental fatigue without adverse effects on safety-related parameters.

## 1. Introduction

The liver is the largest organ involved in nutrient metabolism, detoxification, immunity, as well as hormone metabolism. A relationship between liver health and diet or lifestyle has been consistently reported, and lifestyle modifications, including physical activity and adherence to a balanced diet, are recommended to improve and maintain liver function [1]. The mechanisms and factors involved in liver damage are numerous and diverse, and morphological changes and liver function decline occur due to genetic susceptibility, improper dietary conditions, drug treatment, and alcohol consumption [2]. This causes symptoms such as extreme fatigue, loss of appetite, weakness, and weight loss, as well as serious liver disease [3]. 

A fatty liver is also known as hepatic steatosis, in which fat accumulates abnormally in the liver cells. The consumption of large amounts of fat and cholesterol causes lipid deposition in the liver, leading to hepatomegaly [4]. Chronic liver disease is classified as non-alcoholic fatty liver disease (NAFLD), caused by metabolic abnormalities such as diabetes, obesity, and hyperlipidemia, and alcohol-related liver disease (ALD), caused by alcohol [5,6]. Obesity and alcohol consumption are associated with liver carcinogenesis, and an imbalance between fatty acid synthesis and β-oxidation leads to steatosis in both NAFLD and ALD. Lipid accumulation induces steatosis in NAFLD, whereas ethanol toxicity causes steatosis in ALD [7,8]. Chronic alcohol exposure stimulates the activation of sterol regulatory element binding protein-1c (SREBP-1c) in ALD, which regulates lipid synthesis [9]. NAFLD is associated with a similar impairment in lipid metabolism. In NAFLD, hyperinsulinemia causes the upregulation of SREBP-1c, leading to increased fatty acid synthesis, whereas β-oxidation is reduced [10]. 

Heavy drinking triggers fat accumulation in the hepatocytes, which are damaged by alcohol metabolites. Alcohol metabolism is mainly affected by the oxidative pathway to produce acetaldehyde, including alcohol dehydrogenase (ADH), cytochrome P450 2E1, and catalase [11,12]. In this pathway, these enzymes induce the production of reactive oxygen species (ROS), leading to fatty liver and liver toxicity [13]. Therefore, chronic alcohol exposure induces the accumulation of toxic metabolites in hepatocytes and increases the risk of steatosis. To regulate alcohol-induced lipid accumulation in the liver, natural compounds such as flavonoids, resveratrol, and β-carotene, which conduct antioxidation, anti-inflammation, or improve lipid metabolism, are suggested as potential treatments for ALD [14,15,16]. Vitexin, a natural flavonoid, has a hepatoprotective effect by inhibiting liver lipid accumulation and regulating antioxidant activity, according to a mouse model [17]. Chachay et al. [18] showed that resveratrol supplementation improved glucose and lipid metabolism in NAFLD patients [18]. However, the 8-weeks administration of resveratrol did not reduce insulin resistance or steatosis for NAFLD patients in clinical trials [19]. Zhao et al. [20] showed that lutein attenuated liver injury in chronic ethanol-induced liver and intestinal barrier in rats. Although some natural compounds have been verified, the clinical effect of plant-derived natural compounds is still needed for human studies to validate their function.

*Zizania latifolia*, a wild rice and water bamboo, is widely cultivated in East Asia and is a perennial aquatic plant that uses stems, leaves, and seeds as food materials in Korea. Several recent studies have shown that *Z*. *latifolia* is effective in reducing insulin resistance, suppressing hyperlipidemia, reducing blood glucose levels, exerting antiobesity effects, and preventing skin aging [21,22,23,24]. Lee et al. [25] demonstrated that a methanol extract of *Z. latifolia* exhibits antiallergic effects through the inhibition of compound 48/80-induced degranulation and antigen-induced ß-hexosaminidase release in RBL-2H3 mast cells. Another study reported that one new flavonolignan, one flavone, and three flavonolignans were isolated as the active constituents from the ethyl acetate fraction of the aerial parts of *Z. latifolia*, and that tricin and its derivatives were the main components of *Z. latifolia* [26]. A previous study showed that the enzymatic treatment of a *Z. latifolia* extract increased the tricin content [27]. Consistent with this, ETZL and tricin attenuated UVB-induced skin damage and photoaging in SKH-1 hairless mice and in UVA-irradiated human dermal fibroblasts (HDFs) by inhibiting lysosomal exocytosis and ROS generation [27,28]. Our previous study demonstrated that *Z*. *latifolia* suppressed hepatic lipid accumulation and hepatic damage by upregulating antioxidant defense mechanisms in a binge animal model [29]. However, no clinical studies have reported the hepatic functional improvement of *Z*. *latifolia* after oral intake in humans. Based on previous findings, the present study evaluated whether enzyme-treated *Z*. *latifolia* improves liver-related outcomes and fatigability in a multicenter, randomized, double-blind, placebo-controlled trial. 

## 2. Materials and Methods

### 2.1. Sample Preparation

Enzyme-treated *Z. latifolia* ethanol extract (ETZL) was provided by BTC Corporation (Ansan, Republic of Korea) to evaluate the effect of the tricin (≥92.5%, chromadex, Los Angeles, CA, USA, CAS number 520-32-1)-containing extract on liver function and fatigability. EZTL was prepared as described in a previous study [27]. Briefly, dried leaves of *Z. latifolia* were extracted using mixed hydrolysis enzymes. In addition, enzyme-treated *Z. latifolia* was inactivated by heating. The supernatant was filtered, and the residue was re-extracted using water and ethanol. The combined extract underwent filtration, concentration, and drying to produce ETZL.

Tricin contents of Z. latifolia extract and ETZL were measured and compared using high-performance liquid chromatography (HPLC). Using the HPLC gradient method, water containing 0.15% phosphoric acid and methanol (≥99.9%, Merck KGaA, Darmstadt, Germany) were used as mobile phases, and detailed analysis conditions are shown in Table 1. Tricin standard solutions were prepared at five concentrations ranging from 1 to 20 ppm and used for quantitative analysis, and all analyses were performed in triplicate to verify the validity of the analytical method.

### 2.2. Participants

This study was approved by Wonkwang University (WUJKMH-IRB-2020-0008) and Woosuk University (WSOH IRB H2007-01-01) Korean Medicine Hospital Institutional Review Boards (20 and 27 July 2020) in the Republic of Korea, and was conducted in accordance with the ethical standards of the Declaration of Helsinki (1964). The participants were healthy Korean adults aged 19–60 years, and the study period was from September 2020 to September 2021. Based on the approved protocol, this was a multicenter, randomized, double-blind, placebo-controlled study. All study subjects received a detailed explanation of this clinical trial, voluntarily decided to participate, and gave written consent to comply with the study precautions.

Participants who met the following criteria were included: (1) male and female (19 ≤ age ≤ 60 years); (2) an average alcohol consumption habit of 14 units (1 unit: 10 g of pure alcohol) for men and 7 units for women per week; (3) a serum alanine aminotransferase (ALT) level of 46 U/L or higher and a serum gamma-glutamyl transferase (GGT) level of 65 U/L or higher.

Moreover, participants with the following conditions were excluded: (1) aspartate aminotransferase (AST), ALT, or GGT level >3 times the upper limit or a serum creatinine level >2.0 mg/dL in the diagnostic test; (2) acute and chronic hepatitis, type B, or type C hepatitis virus; (3) a history of alcohol use disorder through the alcohol use disorder identification test; (4) the presence of cirrhosis, liver cancer, or signs of liver cancer; (5) the presence of hepatobiliary disease requiring treatment; (6) body mass index (BMI) <18.5 kg/m^2^ or ≥35 kg/m^2^; (7) the presence of clinically severe cardiovascular, endocrine, neuropsychiatric, musculoskeletal, gastrointestinal, inflammatory, hematological, and/or neoplastic diseases; (8) a history of esophageal varicose bleeding, hepatic coma, ascites, etc., within 1 year before the study’s screening tests; (9) the use of drugs or health-functional foods related to liver function within 1 month before the screening tests; (10) the intake of antipsychotic agents within the previous 3 months; (11) substance abuse; (12) current status of pregnancy or lactation; (13) participation in another clinical trial within 3 months before screening tests; (14) the presence of other reasons of ineligibility as determined by investigators. 

### 2.3. Study Design

A total of 368 volunteers (potential research participants) provided written informed consent, and a screening test was conducted. After the screening tests, a small number of female participants with an average drinking habit of more than 7 units were excluded, and a total of 268 volunteers were excluded from the screening. One hundred male subjects who met all eligibility criteria and were willing to participate in the trial were randomly assigned to the control (*n* = 50) and treatment (*n* = 50) groups. Subject blocks of certain sizes (e.g., 4, 6, and 8) were used for block randomization, and all subjects and investigators were blinded to the randomization sequences until the end of the study. After random assignment, one person each from the control group (*n* = 1; lost to follow-up) and the treatment group (*n* = 1; withdrew consent) was excluded from the analysis according to the full analysis criteria, and efficacy evaluation was conducted on a total of 98 study subjects.

Subjects took capsules containing ETZL (test product) or microcrystalline cellulose (placebo) once daily for 8 weeks. Subjects took 2 capsules orally after breakfast, with a daily intake of 900 mg/day (400 mg/day as ETZL). Each capsule contained excipients, such as microcrystalline cellulose, corn starch, and hydroxypropyl methylcellulose (Table 2). 

### 2.4. Assessment of Physiological Properties

The subjects’ physical characteristics, drinking amount, smoking amount, and physical activity were measured before intake (baseline) and 8 weeks after intake. Physical characteristics include weight, BMI, and waist circumference. To investigate physical activity, metabolic equivalent task (MET) was analyzed using the Global Physical Activity Questionnaire (GPAQ).

### 2.5. Assessment of Efficacy Parameters

An efficacy evaluation analysis was conducted excluding a total of 23 subjects (control group: *n* = 9; treatment group: *n* = 14) who had risk factors affecting liver function. Risk factors that affect liver function included (1) hs-CRP exceeding the upper limit of normal, (2) the presence of gallbladder polyps or kidney cysts according to ultrasound scans, (3) taking non-steroidal anti-inflammatory drugs (NSAIDs) and proton pump inhibitors, and (4) alcohol consumption prior to efficacy assessment visit. 

Efficacy evaluation variables (liver function, lipid metabolism, fatty liver, antioxidant activity, and MFS) were compared between the control (*n* = 40) and treatment (*n* = 35) groups. Indicators of liver function and lipid metabolism were analyzed in the subjects’ sera through the Green Cross Research Institute (Yongin, Korea). Liver function indicators included blood parameters, alanine transaminase (ALT), gamma-glutamyl transferase (GGT), aspartate transaminase (AST), total bilirubin (TBIL), and alkaline phosphatase (ALP). Additionally, lipid metabolism indicators included total cholesterol (TCHO), triglycerides (TGs), high-density lipoprotein cholesterol (HDL-C), and low-density lipoprotein cholesterol (LDL-C). The fatty liver index (FLI) was calculated using BMI, waist circumference, triglycerides, and GGT, and the hepatic steatosis index (HSI) was calculated using ALT, AST, and BMI. As antioxidant indicators, total antioxidant status (TAS) was analyzed using TBA c8000 (TOSHIBA, Tokyo, Japan), and malondialdehyde (MDA) content was analyzed using Agilent 1200 Series (Agilent Technologies, Santa Clara, CA, USA). A multidimensional fatigue scale (MFS) questionnaire developed based on the fatigue assessment inventory was used to assess exercise response [30].

### 2.6. Evaluation of Safety Test

Safety evaluation was performed in the control group (*n* = 49) and treatment group (*n* = 49) on subjects who consumed the test product (or placebo) at least once. To evaluate changes in the body due to taking ETZL or the placebo, adverse reactions and diagnostic tests were performed before intake (baseline) and after intake (8 weeks). Safety biomarkers were analyzed using a Cobas 8000 c702 (Roche, Rotkreuz, Switzerland).

### 2.7. Statistical Analyses

The data are expressed as the mean ± standard deviation, and differences in each group between time points (baseline and at 8 weeks) were assessed using an independent *t*-test and a paired *t*-test. The Chi-squared test was used to check whether two categorical variables were related to each other. The ANCOVA was performed by controlling for baseline non-homogenous efficacy evaluation variables and lifestyle factors as covariates. Based on the criteria determined in the blinded meeting and statistical analysis, an efficacy evaluation was conducted by excluding participants with risk factors affecting liver function. The risk factors included high-sensitivity C-reactive protein (hs-CRP) levels (*n* = 4), gallbladder polyps, renal cysts detected through ultrasound examination (*n* = 9), the use of non-steroidal anti-inflammatory drugs and proton pump inhibitors (*n* = 5), and alcohol consumption before the analysis of efficacy and safety (*n* = 9). Therefore, some subjects were excluded (14 in the test group and 9 in the placebo group), and efficacy evaluation variables were analyzed. Statistical significance was verified at *p* < 0.05, using Statistical Analysis System (version 12.0; SPSS, Inc., Chicago, IL, USA).

## 3. Results

### 3.1. Tricin Analysis

**Figure 1:**
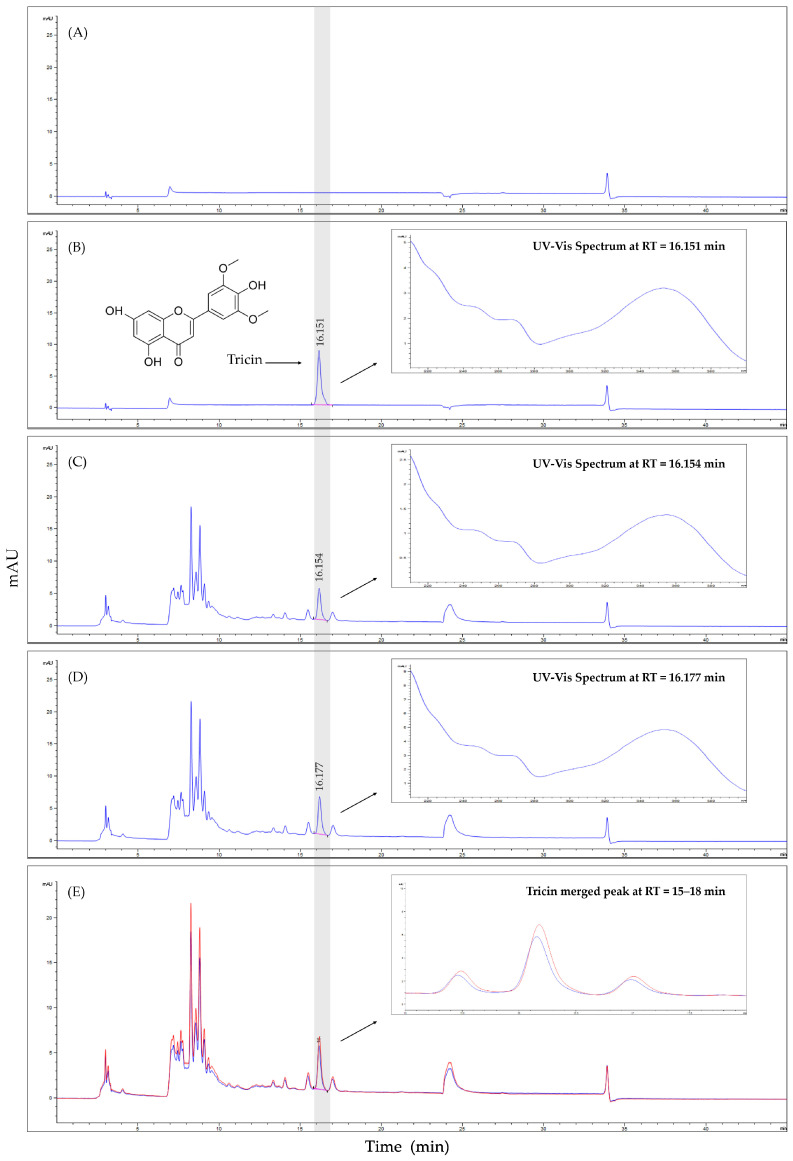
High-performance liquid chromatography (HPLC) chromatogram of tricin content. (**A**) HPLC chromatogram of blank, (**B**) HPLC chromatogram and UV–Vis spectrum of tricin (standard), (**C**) HPLC chromatogram and UV–Vis spectrum of *Z. latifolia* extract, (**D**) HPLC chromatogram and UV–Vis spectrum of Enzyme-treated *Z. latifolia* extract (ETZL), and (**E**) Tricin peaks of merged *Z. latifolia* extract (blue) and ETZL (red line) on HPLC chromatogram.

Figure 1 shows HPLC chromatograms of *Z. latifolia* extract and ETZL. *Z. latifolia* extract contains 0.72 ± 0.03 mg/g of tricin. The tricin content of ETZL was 0.95 ± 0.05 mg/g, and enzyme treatment increased the tricin content. The calibration curve of the tricin standard was y = 35.5413x − 3.3183 and R-squared (R^2^) = 1, showing excellent linearity. Additionally, the detection limit and quantification limit were 0.0211 and 0.0639 μg/mL, respectively.

### 3.2. Demographic Characteristics of the Participants

The demographic characteristics of the study subjects are shown in Table 3. The average age of the subjects was 39.89 ± 8.64 years (control group: 39.68 ± 9.15 years; treatment group: 40.10 ± 8.18 years); no statistically significant difference was observed in age between the two groups. There were no significant differences in body weight, height, BMI, BMR, SBP, DBP, or pulse rate between the control and treatment groups. The average quantity of alcohol consumption was 26.73 ± 12.19 units/week (in which one unit equals 10 g of pure alcohol). The 56 smokers (control group: 27; treatment group: 29) included in this study, on average, smoked 14.61 ± 6.91 cigarettes/day; no significant difference was noted between the two groups with regard to smoking or alcohol consumption.

### 3.3. Effects of ETZL on Physical Activity

A comparison of anthropometric measurements (body weight, BMI, and waist circumference) at baseline and after 8 weeks revealed no statistical changes in either group (Table 4). The amount of physical activity (MET) was also measured at baseline and after 8 weeks, but no statistically significant difference occurred between the two groups (Table 4). In addition, we confirmed the alcohol consumption and smoking amount between baseline and week 8 in both groups (Table 4). A statistically significant difference was found in alcohol consumption (units/week) between baseline and week 8 in each group (*p* = 0.0001); however, no significant difference was found between the groups.

### 3.4. Effects of ETZL on Liver Function

Aminotransferases, including ALT and AST, are important indicators for the diagnosis of liver and biliary tract diseases. As shown in Table 5, the ALT level was significantly lower in the treatment group (*p* = 0.004) after 8 weeks of treatment than at baseline and was significantly different between the groups (*p* = 0.037). The AST/ALT ratio was significantly higher in the treatment group (*p* = 0.001) than at baseline after 8 weeks; however, no statistically significant difference was observed between the two groups. No significant changes were noted in GGT, AST, ALP, or total bilirubin levels before and after 8 weeks of treatment in either group or between the two groups. 

### 3.5. Effects of ETZL on Lipid Metabolism

As shown in Table 6, HDL-C levels were significantly higher in the treatment group (*p* = 0.028) after 8 weeks of treatment than at baseline. However, no statistically significant differences were observed between the two groups. In addition, no significant differences in TCHO, TG, or LDL-C levels were observed between the two groups. The FLI of the treatment group was lower after 8 weeks than that at baseline, but no significant difference was found between the groups. The HSI level was significantly lower in the treatment group (*p* = 0.004) after 8 weeks of treatment than at baseline, with a significant difference between the two groups (*p* = 0.042). 

### 3.6. Effects of ETZL on Antioxidant Activity

The antioxidant activity results are presented in Table 7. The TAS level did not show a significant difference between baseline and week 8 in either group, and no difference occurred between the groups. MDA, a marker of oxidative stress and an end product of lipid peroxidation, was lower than baseline in both the control (*p* = 0.022) and treatment groups after 8 weeks of treatment; however, no significant difference occurred between the two groups. 

### 3.7. Effects of ETZL on Multidimensional Fatigue Scale

As shown in Table 8, MFS, including general, physical, temporal, and total fatigue, was measured. The general and total fatigue scores of the treatment group were significantly lower after 8 weeks of treatment than at baseline (*p* = 0.012 and *p* = 0.032, respectively). However, no significant change occurred in MFS score in the control group. 

### 3.8. Effects of ETZL on Safety Parameters

Blood and urine samples were analyzed to assess the safety of the 98 participants who provided written informed consent (Table 9). A statistically significant difference was observed in blood creatinine levels between the two intake groups (*p* = 0.006); however, this change was not clinically significant within the reference range. There were no statistically significant differences in other parameters between the two groups. Although an adverse reaction was reported in one participant (control group, *n* = 1) during the study period, there was no significant difference in the incidence of adverse reactions between the two groups (*p* > 0.05). The adverse reaction was one case of nasal cold, which was considered to have no clear causal relationship with the intake of the test product.

## 4. Discussion

This clinical trial reported the effect of *Z. latifolia* on liver-related outcomes and fatigue. The stems, roots, and grains of *Z. latifolia*, are widely used as food, supplements, and medicines to inhibit lipid accumulation in the liver, prevent atherogenesis, and promote good health [23,31,32]. A recent study showed that ETZL suppresses hepatic lipid accumulation and hepatic damage through Nrf2 activation in a binge drinking model [29]. Several studies have shown that the extract of *Z. latifolia* has large amounts of proanthocyanidins, flavonoids, and phenolics, and exerts a significant antioxidant effect [33,34]. These findings suggest that *Z. latifolia* exerts therapeutic effects against various diseases.

Blood levels of GGT, ALT, and AST are known markers of liver injury. GGT, which is a sensitive marker of hepatobiliary diseases, is found in liver cells. When the liver cell membrane is damaged, blood AST and ALT levels increase with hepatocellular injury [35,36]. The dominance of AST over ALT is related to alcoholic liver disease and increased mitochondrial aspartate activity owing to mitochondrial damage in heavy drinkers. Several studies have confirmed that antioxidants and anti-inflammatory agents regulate oxidative stress in liver diseases [37]. Chen et al. reported that diallyl trisulfide (DAT), which is a bioactive compound from garlic, decreased serum levels of AST and ALT by inhibiting oxidative stress and apoptosis in alcohol-induced liver injury [38]. Similar findings reported by Mirhafez et al. showed that daily supplementation with low-dose curcumin for 2 months substantially reduced serum AST levels in patients with NAFLD compared with the placebo group [39]. 

The liver plays a major role in the distribution of lipids to the other organs. In the liver, fatty acids are converted to triglycerides and cholesterol esters, which are then secreted as VLDL [40]. Imbalances in lipid metabolism in the liver can induce steatosis or the accumulation of triglycerides in hepatocytes [41]. Zhang et al. reported that consuming *Z. latifolia* decreased serum TAG and total cholesterol levels and increased HDL cholesterol levels in rats fed a high-fat/cholesterol diet [24]. In addition, ETZL treatment regulated lipid metabolism genes such as SREBP-1c, FASN, ACC, and PPAR in an NAFLD model [21,23]. The present study showed that ETZL treatment increased HDL cholesterol levels and decreased LDL cholesterol levels (Table 3). 

The results obtained in this study suggest that 8 weeks of ETZL administration reduced TAS and MDA levels (Table 4). As oxidative stress is involved in the pathogenesis of various liver diseases, the measurement of antioxidant activity has been suggested as a screening tool for hepatic damage. MDA is known as one of the major end products of lipid peroxidation [42]. Therefore, the alcohol-induced lipid peroxide content in tissues can be measured using the amount of MDA. Alcohol consumption results in a substantial increase in liver MDA content compared to without consumption [43]. Selim et al. [44] showed that *Plumbago indica*, a dicotyledonous plant, exerted marked hepatoprotective effects against thioacetamide-induced liver oxidative stress and fibrosis in rats by reducing MDA levels. Our previous study reported that ETZL decreased the serum levels of ALT, AST, TG, and MDA in binge-drinking rats by upregulating antioxidant defense mechanisms [29]. 

This study measured multidimensional fatigue to examine whether ETZL treatment regulated fatigue. Changes in the multidimensional fatigue scale were observed in the ETZL treatment group compared with that in the control group (Table 5). Fatigue is the most common symptom in patients with liver disease and has a strong impact on their quality of life [45]. Mental (central) fatigue is characterized by a lack of self-motivation for physical and mental activities. Physical (peripheral) fatigue is characterized by neuromuscular and muscle weakness [46,47]. Swain et al. demonstrated that patients with CLD exhibit both dimensions of fatigue [45]. Because the liver regulates the storage, release, and production of substrates for energy generation, understanding the pathogenesis of fatigue in the liver may be a helpful approach for treating patients with liver disease. Kim et al. showed that the administration of the aerial parts of *Z. latifola* for 14 days significantly decreased the immobility times in a forced swimming test in mice [48]. Therefore, *Z. latifola* is considered a potential therapeutic agent for liver-disease-associated fatigue.

## 5. Conclusions

The results showed that the administration of ETZL containing tricin for 8 weeks altered the levels of liver enzymes, lipid metabolism, and antioxidant activity compared to the control group. Moreover, ETZL induced changes in overall fatigue when comparing the baseline and 8 weeks of treatment. Based on these findings, ETZL, when administered orally, may act as a hepatoprotective agent to improve liver function. However, further studies involving long-term supplementation with ETZL and the pharmacokinetics of tricin are required to confirm these results.

## Figures and Tables

**Table 1:** Analytical conditions of high-performance liquid chromatography.

Parameters	Condition		
Instrument	Agilent Infinity 1200 series		
Column	SUPELCO Discovery C18 column (4.6 × 250 mm, 5 µm)		
Column temperature	30 °C		
Injection volume	10 µL		
Flow rate	1.0 mL/min		
Detector wavelength	350 nm		
Mobile phase	A: 0.15% phosphoric acid in water B: methanol		
Gradient	Time (min)	A (%)	B (%)
	0	80	20
	3	80	20
	8	50	50
	20	45	55
	30	15	85
	45	80	20

**Table 2:** Description of the placebo and treatment products.

Ingredients	Contents (%)	
	Placebo Product	Treatment Product
Enzyme-treated *Zizania latifolia* extract	-	44.44
Microcrystalline cellulose	87.00	45.55
Corn starch	3.00	3.00
Hydroxypropyl methylcellulose	2.00	2.00
Beta-cyclodextrin	1.50	1.50
Silicon dioxide	1.50	1.50
Magnesium stearate	1.00	1.00
Sodium carboxymethyl cellulose	1.00	1.00
Gardenia yellow color	1.50	-
Cacao color	1.50	-
Features	Yellowish gray capsule	
Packing	One capsule (450 mg) individually packed	

**Table 3:** Demographic characteristics of the participants.

	Control Group	Treatment Group	Total (*n* = 100)	*p*-Value ^(1)^
Sex (male/female)	50/0	50/0	100/0	
Age (years)	39.68 ± 9.15	40.10 ± 8.18	39.89 ± 8.64	0.809
Height (cm)	174.64 ± 6.58	174.56 ± 5.98	174.60 ± 6.26	0.949
Weight (kg)	87.88 ± 14.34	88.07 ± 14.53	87.97 ± 14.36	0.949
BMI (kg/m^2^)	28.67 ± 3.35	28.79 ± 3.88	28.73 ± 3.60	0.862
Waist circumference (cm)	95.92 ± 8.74	95.07 ± 10.16	95.50 ± 9.44	0.656
SBP (mmHg)	129.72 ± 9.23	131.26 ± 11.05	130.49 ± 10.16	0.451
DBP (mmHg)	82.82 ± 7.05	83.68 ± 8.25	83.25 ± 7.76	0.576
Pulse (times/min)	77.70 ± 9.11	76.84 ± 11.45	77.27 ± 10.30	0.679
AFP (ng/mL)	3.07 ± 2.27	3.55 ± 1.93	3.31 ± 2.11	0.253
HBsAg (IU/L)	0.41 ± 0.09	0.39 ± 0.07	0.40 ± 0.08	0.502
Anti-HBs (IU/L)	151.90 ± 270.09	190.36 ± 329.50	171.13 ± 300.36	0.525
Anti-HBc IgM (COI)	0.08 ± 0.03	0.07 ± 0.01	0.08 ± 0.02	0.216
Anti-HCV (S/CO)	0.07 ± 0.04	0.16 ± 0.52	0.12 ± 0.37	0.218
Alcohol (units/week)	24.92 ± 11.54	28.53 ± 12.67	26.73 ± 12.19	0.140
Alcohol (units/day)	0.23 ± 0.98	0.64 ± 2.42	0.44 ± 1.85	0.272
Smoking (*n*, %)	27 (54.00%)	29 (58.00)	56 (56%)	0.687 ^(2)^

Values are presented as the mean ± standard deviation. *p*-value ^(1)^ was analyzed with an independent *t*-test between groups, and *p*-value ^(2)^ was analyzed with a Chi-squared test between groups. BMI, body mass index; SBP, systolic blood pressure; DBP, diastolic blood pressure; AFP, alpha-fetoprotein; HBsAg, hepatitis B virus surface antigen; HBc IgM, hepatitis B core IgM; HCV, hepatitis C virus.

**Table 4:** Changes in physical activity between baseline and week 8.

Indicators	Control Group (*n* = 49)				Treatment Group (*n* = 49)				*p*-Value ^(2)^
	Baseline	8 Weeks	Diff	*p*-Value ^(1)^	Baseline	8 Weeks	Diff	*p*-Value ^(1)^	
Weight (kg)	88.02 ± 14.22	88.54 ± 14.22	0.52 ± 1.88	0.061	88.02 ± 14.67	87.92 ± 15.22	−0.10 ± 2.05	0.740	0.129
BMI (kg/m^2^)	28.68 ± 3.38	28.84 ± 3.25	0.17 ± 0.62	0.066	28.80 ± 3.92	28.76 ± 4.10	−0.04 ± 0.69	0.649	0.112
WC (cm)	96.02 ± 8.80	96.80 ± 8.75	0.78 ± 2.61	0.041 *	94.99 ± 10.25	95.31 ± 10.61	0.31 ± 3.37	0.516	0.442
MET (min/week)	1666.94 ± 1821.25	1638.78 ± 2950.88	−28.16 ± 2312.48	0.932	1782.04 ± 2386.03	1737.96 ± 3225.31	−44.08 ± 3232.27	0.924	0.978
Alcohol (units/week)	24.96 ± 11.66	18.25 ± 10.20	−6.71 ± 9.28	0.0001 ***	28.64 ± 12.77	22.38 ± 12.95	−6.27 ± 8.26	0.0001 ***	0.802
Alcohol (units/day)	0.12 ± 0.57	0.00 ± 0.00	−0.12 ± 0.57	0.160	0.53 ± 2.33	0.25 ± 0.91	−0.29 ± 2.48	0.424	0.643
Smoking (cigarette/day)	15.23 ± 7.88	14.58 ± 8.13	−0.65 ± 2.04	0.115	13.86 ± 6.04	13.10 ± 5.55	−0.78 ± 2.21	0.078	0.833

Values are presented as the mean ± standard deviation. *p*-value ^(1)^ was analyzed with an independent *t*-test between groups, and *p*-value ^(2)^ was analyzed with a Chi-squared test between groups. Symbols indicate significant differences at * *p* < 0.05 and *** *p* < 0.001. BMI: body mass index; WC: waist circumference; MET: metabolic equivalent task.

**Table 5:** Effects of enzyme-treated *Zizania latifolia* ethanol extract on liver function.

Indicators	Control Group (*n* = 40)				Treatment Group (*n* = 35)				*p*-Value ^(2)^
	Baseline	8 Weeks	Diff	*p*-Value ^(1)^	Baseline	8 Weeks	Diff	*p*-Value ^(1)^	
ALT (U/L)	69.70 ± 24.14	68.70 ± 30.92	−1.00 ± 24.71	0.799	70.20 ± 26.61	56.86 ± 29.01	−13.34 ± 25.59	0.004 **	0.037 *
GGT (U/L)	94.30 ± 31.62	91.70 ± 47.48	−2.60 ± 36.15	0.652	113.03 ± 41.31	101.77 ± 47.95	−11.26 ± 36.69	0.078	0.308
AST (U/L)	40.85 ± 14.02	41.20 ± 14.50	0.35 ± 11.96	0.854	41.03 ± 15.36	36.34 ± 14.62	−4.69 ± 15.28	0.078	0.114
ALP (U/L)	108.35 ± 79.31	111.83 ± 78.46	3.48 ± 17.62	0.220	106.63 ± 61.36	106.54 ± 59.48	−0.09 ± 15.47	0.974	0.359
TBIL (mg/dL)	0.92 ± 0.33	0.95 ± 0.29	0.03 ± 0.31	0.553	0.86 ± 0.28	0.94 ± 0.30	0.08 ± 0.25	0.077	0.460
AST/ALT ratio	0.60 ± 0.15	0.67 ± 0.34	0.07 ± 0.25	0.088	0.61 ± 0.18	0.72 ± 0.26	0.11 ± 0.18	0.001 **	0.410

Values are presented as the mean ± standard deviation. *p*-value ^(1)^ was analyzed with an independent *t*-test between groups, and *p*-value ^(2)^ was analyzed with a Chi-squared test between groups. Symbols indicate significant differences at * *p* < 0.05 and ** *p* < 0.01. ALT, alanine transaminase; GGT, gamma-glutamyl transferase; AST, aspartate transaminase; ALP, alkaline phosphatase; TBIL, total bilirubin.

**Table 6:** Effects of enzyme-treated *Zizania latifolia* ethanol extract on lipid metabolism.

Indicators	Control Group (*n* = 40)				Treatment Group (*n* = 35)				*p*-Value ^(2)^
	Baseline	8 Weeks	Diff	*p*-Value ^(1)^	Baseline	8 Weeks	Diff	*p*-Value ^(1)^	
TCHO (mg/dL)	229.00 ± 42.85	226.85 ± 38.22	−2.15 ± 29.01	0.642	227.97 ± 44.71	228.57 ± 52.27	0.60 ± 39.08	0.928	0.728
TG (mg/dL)	215.13 ± 82.37	217.23 ± 92.88	2.10 ± 102.52	0.898	230.86 ± 83.74	243.86 ± 156.15	13.00 ± 134.81	0.572	0.693
HDL-C (mg/dL)	48.33 ± 12.20	49.13 ± 10.99	0.80 ± 6.96	0.472	50.14 ± 14.61	54.46 ± 14.66	4.31 ± 11.13	0.028 *	0.113
LDL-C (mg/dL)	142.95 ± 38.54	145.70 ± 41.29	2.75 ± 23.43	0.462	139.26 ± 42.68	135.26 ± 45.49	−4.00 ± 31.78	0.462	0.295
FLI	78.03 ± 16.28	78.43 ± 15.04	0.40 ± 9.21	0.783	78.37 ± 18.64	74.38 ± 23.61	−4.00 ± 12.77	0.073	0.096
HSI	42.36 ± 5.00	41.94 ± 5.84	−0.42 ± 2.65	0.325	42.59 ± 6.83	40.67 ± 7.03	−1.92 ± 3.62	0.004 **	0.042 *

Values are presented as the mean ± standard deviation. *p*-value ^(1)^ was analyzed with an independent *t*-test between groups, and *p*-value ^(2)^ was analyzed with a Chi-squared test between groups. Symbols indicate significant differences at * *p* < 0.05 and ** *p* < 0.01. TCHO, total cholesterol; TGs, triglycerides; HDL-C, high-density lipoprotein cholesterol; LDL-C, low-density lipoprotein cholesterol; FLI, fatty liver index; HSI, hepatic steatosis index.

**Table 7:** Effects of enzyme-treated *Zizania latifolia* ethanol extract on antioxidant activity.

Indicators	Control Group (*n* = 40)				Treatment Group (*n* = 35)				*p*-Value ^(2)^
	Baseline	8 Weeks	Diff	*p*-Value ^(1)^	Baseline	8 Weeks	Diff	*p*-Value ^(1)^	
TAS (nmol/L)	1.87 ± 0.26	1.86 ± 0.26	−0.02 ± 0.27	0.647	1.89 ± 0.22	1.88 ± 0.26	−0.01 ± 0.26	0.897	0.818
MDA (pmol/mL)	1801.00 ± 1174.93	1382.00 ± 399.28	−419.00 ± 1106.24	0.022 *	1546.29 ± 472.35	1485.14 ± 483.74	−61.14 ± 342.44	0.298	0.058

Values are presented as the mean ± standard deviation. *p*-value ^(1)^ was analyzed with an independent *t*-test between groups, and *p*-value ^(2)^ was analyzed with a Chi-squared test between groups. Symbols indicate significant differences at * *p* < 0.05.

**Table 8:** Effects of enzyme-treated *Zizania latifolia* ethanol extract on multidimensional fatigue scale.

Indicators	Control Group (*n* = 40)				Treatment Group (*n* = 35)				*p*-Value ^(2)^
	Baseline	8 Weeks	Diff	*p*-Value ^(1)^	Baseline	8 Weeks	Diff	*p*-Value ^(1)^	
General fatigue	26.30 ± 11.35	26.15 ± 11.14	−0.15 ± 7.28	0.897	24.80 ± 8.85	21.74 ± 9.88	−3.06 ± 6.84	0.012 *	0.080
Physical fatigue	21.48 ± 7.15	21.33 ± 7.09	−0.15 ± 4.81	0.845	20.77 ± 6.24	19.66 ± 6.51	−1.11 ± 5.35	0.226	0.414
Temporal fatigue	18.70 ± 5.61	18.78 ± 6.75	0.08 ± 5.01	0.925	17.77 ± 5.96	16.66 ± 6.63	−1.11 ± 5.06	0.202	0.311
Total fatigue	66.48 ± 21.71	66.25 ± 23.20	−0.23 ± 13.79	0.918	63.34 ± 19.35	58.06 ± 21.34	−5.29 ± 13.96	0.032 *	0.119

Values are presented as the mean ± standard deviation. *p*-value ^(1)^ was analyzed with an independent t-test between groups, and *p*-value ^(2)^ was analyzed with a Chi-squared test between groups. Symbols indicate significant differences at * *p* < 0.05.

**Table 9:** Effects of enzyme-treated *Zizania latifolia* ethanol extract on safety biomarkers.

Indicators		Control Group (*n* = 49)				Treatment Group (*n* = 49)				*p*-Value ^(2)^
		Baseline	8 Weeks	Diff	*p*-Value ^(1)^	Baseline	8 Weeks	Diff	*p*-Value ^(1)^	
Complete blood count	WBC (×10^3^/µL)	7.22 ± 1.62	7.07 ± 1.74	−0.15 ± 1.21	0.379	7.37 ± 1.63	6.87 ± 1.78	−50 ± 1.38	0.014 *	0.185
	RBC (×100^3^/µL)	5.15 ± 0.42	5.09 ± 0.41	−0.05 ± 0.22	0.104	5.08 ± 0.32	5.05 ± 0.40	−0.03 ± 0.27	0.483	0.616
	Hb (g/dL)	15.98 ± 0.95	15.90 ± 0.96	−0.08 ± 0.58	0.333	15.83 ± 0.74	15.74 ± 1.06	−0.08 ± 0.78	0.451	0.978
	Hct (%)	47.75 ± 2.91	47.68 ± 3.20	−0.07 ± 2.05	0.809	47.31 ± 2.26	47.41 ± 3.27	0.09 ± 2.95	0.827	0.750
	Platelet (×10^3^/µL)	253.94 ± 57.74	245.58 ± 51.42	−8.36 ± 34.91	0.097	256.06 ± 44.76	255.58 ± 56.16	−0.48 ± 29.26	0.909	0.224
Serum Biochemistry	Total protein (g/dL)	7.26 ± 0.36	7.39 ± 0.40	0.12 ± 0.52	0.095	7.28 ± 0.41	7.35 ± 0.40	0.07 ± 0.51	0.334	0.599
	Albumin (g/dL)	4.37 ± 0.30	4.44 ± 0.29	0.07 ± 0.38	0.199	4.43 ± 0.33	4.53 ± 0.32	0.10 ± 0.39	0.073	0.697
	BUN (mg/dL)	13.67 ± 2.99	13.78 ± 3.32	0.11 ± 2.98	0.799	13.25 ± 2.99	13.4 ± 3.31	0.16 ± 3.09	0.723	0.937
	Creatine (mg/dL)	0.99 ± 0.14	1.02 ± 0.14	0.03 ± 0.10	0.034 *	0.98 ± 0.12	0.97 ± 0.13	−0.02 ± 0.07	0.071	0.006 **
	Glucose (mg/dL)	108.54 ± 13.20	107.48 ± 14.19	−1.06 ± 12.93	0.565	106.96 ± 12.59	108.00 ± 16.70	1.04 ± 13.11	0.577	0.422
	CK (U/L)	131.76 ± 73.98	143.84 ± 78.30	12.08 ± 69.56	0.225	171.12 ± 226.35	129.38 ± 61.93	−41.74 ± 205.15	0.157	0.084
	LDH (U/L)	180.78 ± 33.79	184.56 ± 27.66	3.78 ± 27.81	0.341	183.64 ± 32.66	185.72 ± 35.09	2.08 ± 28.28	0.605	0.762
	hs-CRP (mg/L)	1.22 ± 1.11	1.75 ± 2.12	0.53 ± 2.01	0.066	1.42 ± 1.17	1.54 ± 1.53	0.12 ± 1.43	0.562	0.236
Urinalysis	Specific gravity	1.03 ± 0.01	1.03 ± 0.01	0.00 ± 0.00	0.728	1.02 ± 0.01	1.03 ± 0.00	0.00 ± 0.01	0.136	0.130
	pH	5.71 ± 0.57	5.67 ± 0.56	−0.04 ± 0.60	0.637	5.81 ± 0.72	5.83 ± 0.62	0.02 ± 0.81	0.848	0.664

Values are presented as the mean ± standard deviation. *p*-value ^(1)^ was analyzed with an independent *t*-test between groups, and *p*-value ^(2)^ was analyzed with a Chi-squared test between groups. Symbols indicate significant differences at * *p* < 0.05 and ** *p* < 0.01. WBC, white blood cell; RBC, red blood cell; Hb, hemoglobin; Hct, hematocrit; BUN, blood urea nitrogen; CK, creatine kinase; LDH, lactate dehydrogenase; hs-CRP, high-sensitivity C-reactive protein.

## Data Availability

The original contributions presented in the study are included in the article, further inquiries can be directed to the corresponding authors.
