# Correction: Ahn et al. Enzyme-Treated *Zizania latifolia* Ethanol Extract Improves Liver-Related Outcomes and Fatigability. *Foods* 2024, *13*, 1725

**DOI:** 10.3390/foods13152362

**Published:** 2024-07-26

**Authors:** Yu-Jin Ahn, Boyun Kim, Yoon Hee Kim, Tae Young Kim, Hyeyeong Seo, Yooheon Park, Sung-Soo Park, Yejin Ahn

**Affiliations:** 1 Dental Research Institute, School of Dentistry, Seoul National University, Seoul 03080, Republic of Korea; yuahn@snu.ac.kr; 2 Department of Smart-Bio, Kyungsung University, Busan 48434, Republic of Korea; boyunism@gmail.com; 3 R&D Center, BTC Corporation, Ansan 15588, Republic of Korea; kyh@btcbio.com (Y.H.K.); tykim@btcbio.com (T.Y.K.); 4 Department of Food Science and Biotechnology, Dongguk University, Goyang 10326, Republic of Korea; hyeyeong09@dongguk.edu (H.S.); ypark@dongguk.edu (Y.P.); 5 Department of Food Science and Nutrition, Jeju National University, Jeju 63243, Republic of Korea; 6 Research Group of Functional Food Materials, Korea Food Research Institute, Wanju-gun 55365, Republic of Korea

## Error in Table

In the original publication [1], there was a mistake in Table 1 as published. The corrected Table 1 appears below. 

## Text Correction

There was an error in the original publication. A correction has been made to Section 2. Materials and Methods, 2.1. Sample Preparation:

Tricin contents of *Z. latifolia* extract and ETZL were measured and compared using high-performance liquid chromatography (HPLC). Using the HPLC gradient method, water containing 0.15% phosphoric acid and methanol (≥99.9%, Merck KGaA, Darmstadt, Germany) were used as mobile phases, and detailed analysis conditions are shown in Table 1. Tricin standard solutions were prepared at five concentrations ranging from 1 to 20 ppm and used for quantitative analysis, and all analyses were performed in triplicate to verify the validity of the analytical method.

The authors state that the scientific conclusions are unaffected. This correction was approved by the Academic Editor. The original publication has also been updated.

## Figures and Tables

**Table 1:** Analytical conditions of high-performance liquid chromatography.

Parameters	Condition		
Instrument	Agilent Infinity 1200 series		
Column	SUPELCO Discovery C18 column (4.6 × 250 mm, 5 μm)		
Column temperature	30 °C		
Injection volume	10 μL		
Flow rate	1.0 mL/min		
Detector wavelength	350 nm		
Mobile phase	A: 0.15% phosphoric acid in water B: methanol		
Gradient	Time (min)	A (%)	B (%)
	0	80	20
	3	80	20
	8	50	50
	20	45	55
	30	15	85
	45	80	20

## References

[1] Ahn Y.-J., Kim B., Kim Y.H., Kim T.Y., Seo H., Park Y., Park S.-S., Ahn Y. (2024). Enzyme-Treated *Zizania latifolia* Ethanol Extract Improves Liver-Related Outcomes and Fatigability. Foods.

